# Experimental herbivore exclusion, shrub introduction, and carbon sequestration in alpine plant communities

**DOI:** 10.1186/s12898-018-0185-9

**Published:** 2018-08-30

**Authors:** Mia Vedel Sørensen, Bente Jessen Graae, Dagmar Hagen, Brian J. Enquist, Kristin Odden Nystuen, Richard Strimbeck

**Affiliations:** 1 Department of Biology, NTNU, Norwegian University of Science and Technology, Høgskoleringen 5, 7491 Trondheim, Norway; 2 Faculty of Biosciences and Aquaculture, Nord University, P.O. Box 2501, 7729 Steinkjer, Norway; 3 Department of Ecology and Evolutionary Biology, University of Arizona, BioSciences West, Tucson, AZ 85721 USA; 4 The Santa Fe Institute, 1399 Hyde Park Rd, Santa Fe, NM 87501 USA; 5 Norwegian Institute for Nature Research, P.O. Box 5685, Torgarden, 7485 Trondheim, Norway

**Keywords:** Gross ecosystem photosynthesis, Ecosystem respiration, Salix, Grazing, Tundra, Meadow, Heath, Shrub expansion, Herbivory, Biomass

## Abstract

**Background:**

Shrub cover in arctic and alpine ecosystems has increased in recent decades, and is predicted to further increase with climate change. Changes in shrub abundance may alter ecosystem carbon (C) sequestration and storage, with potential positive feedback on global C cycling. Small and large herbivores may reduce shrub expansion and thereby counteract the positive feedback on C cycling, but herbivore pressures have also changed in the alpine-arctic tundra; the increased shrub cover together with changes in herbivore pressure is leading to unpredictable changes in carbon sequestration and storage. In this study we investigate the importance of herbivory and shrub introduction for carbon sequestration in the short term. We measured standing biomass and daytime mid-growing season carbon fluxes in plots in a full factorial design where we excluded small and large mammalian herbivores and introduced *Salix* by planting *Salix* transplants. We used three study sites: one *Empetrum*-dominated heath, one herb- and cryptogam-dominated meadow, and one *Salix*-dominated shrub community in the low-alpine zone of the Dovre Mountains, Central Norway.

**Results:**

After 2 years, significant treatment effects were recorded in the heath community, but not in the meadow and shrub communities. In the heath community cessation of herbivory increased standing biomass due to increased biomass of dwarf shrubs. Cessation of herbivory also reduced biomass of bryophytes and ecosystem respiration (ER). Except for an increase in biomass of deciduous shrubs caused by the *Salix* introduction, the only effect of *Salix* introduction was an increase in biomass of graminoids in the heath.

**Conclusions:**

Our short-term study demonstrated that herbivore exclusion had small but still significant effects on heath vegetation, whereas such effects were not apparent in the herb-and cryptogam-dominated meadow and the *Salix*-dominated shrub community. Following the treatments over more years is needed to estimate the long-term effects on community structure and the consequences for C sequestration in the three plant communities. Such data are important for predicting the impact of shrub expansion on C budgets from alpine regions.

**Electronic supplementary material:**

The online version of this article (10.1186/s12898-018-0185-9) contains supplementary material, which is available to authorized users.

## Background

High-latitude alpine and arctic tundra ecosystems are very important for global carbon sequestration, as they currently store more than half of global soil carbon (C) [1]. The large C accumulation is due to short growing seasons, and low rates of decomposition caused by low temperatures, waterlogging, and permafrost [2]. These high-latitude ecosystems are extremely sensitive to climate changes with projections of some of the greatest C losses [3]. In recent decades, shrub cover in circumpolar arctic and alpine tundra ecosystems has increased [4–8], and climate change and reduced herbivory have been proposed as the main reasons for shrub expansion [9–16]. The taller statured shrubs increase biomass and above-ground carbon storage, and may lead to higher net summer CO_2_ sequestration [17]. Therefore, modeling studies predict shrub expansion to increase ecosystem carbon sequestration [18–20]. On the other hand, field studies suggest total ecosystem carbon storage may decline, because shrub expansion may reduce soil carbon storage, and cause increased rates of decomposition and higher ecosystem respiration [17, 21–25]. Hence, more knowledge of these processes is needed, since shrub expansion can potentially alter ecosystem C cycling with positive feedback to the atmosphere if plant uptake of C is lower than the amount of soil C released.

Herbivores affect vegetation composition and ecosystem structure [26, 27] either by consumption, trampling or by adding N via excretion [28]. Herbivores reduce tall deciduous shrub growth, and maintain low-growing tundra vegetation [15, 29, 30], and may also decrease radial growth [31]. Herbivores could therefore counteract the carbon cycle effects of shrub expansion by reducing above-ground biomass and decreasing rates of C cycling [14, 32–35]. Still, there is not much consensus on ecosystem C sequestration and storage consequences of herbivory exclusion [32, 34–41]. Regarding gross ecosystem photosynthesis (GEP), most previous studies found herbivory decreased carbon fixed by the vegetation [32, 34, 42, 43]. Yet, other studies found no difference in GEP with herbivory [44] or an increase over a 50 years experiment due to changes in plant community composition [39]. Ecosystem respiration (ER) has been reported not to differ with grazing and browsing [32, 34, 37, 39, 44] or to decrease [38, 42, 45]. However, one study found increased ER with heavy grazing of reindeers, as compared to lightly grazed plots [43]. Grazing can also promote root exudation, which stimulates microbial activity and thereby increases heterotrophic respiration [35, 46]. While a meta-analysis found that herbivores decrease soil respiration in the subarctic, sheep presence in temperate grasslands can increase soil respiration [38]. These opposing results of carbon sequestration in alpine and arctic ecosystems are due to variation in the ecosystem effects of herbivory with plant community, herbivore species, herbivore pressure, and temporal and spatial scale of the experiment [35, 39, 41, 47].

Herbivore pressure in arctic and alpine ecosystems has changed over the past decades. In alpine areas of Norway, land use changes involving structural changes in husbandry and abandonment of summer pastures has increased the presence of browsing cervids (e.g., reindeer and moose) and decreased browsing and grazing livestock (sheep) [48]. However certain areas of Norway experience locally higher browsing and grazing pressure than before due to bigger herds [49]. Population cycles of small herbivores such as voles and lemmings have also changed with tendencies for collapses in recent decades [50, 51]. In Norway, ptarmigan populations have also declined [52]. Because of the variation in herbivore densities, it is important to understand both large and small herbivore impact on community structure, shrub expansion, and C cycling [10, 13, 26, 51].

Variation in snow-depth and nutrient and moisture conditions creates mosaics of vegetation types in the alpine and arctic tundra [53, 54]. Dwarf shrub-dominated heath and meadow are common vegetation types in alpine and arctic tundra that are vulnerable to shrub expansion under climate change [55, 56]. Since meadow and heath communities are subject to shrub expansion, we experimentally put out *Salix* transplants for shrub introduction into those two communities. *Salix* transplants have been used in alpine nature restoration, with results such as increased total biomass and lateral growth of the *Salix* after two growing seasons [57]. Still, *Salix* establishment in closed vegetation may be very slow compared to establishment on bare soil [57]. Direct introduction provides a novel method to study the changes likely to happen with future shrub expansion in in these plant communities, and here we provide important baseline data. To investigate the importance of shrub expansion and herbivory for ecosystem functioning, we excluded small and large mammalian herbivores and introduced *Salix* with *Salix* transplants in a full factorial design with plots in *Empetrum*-dominated dwarf shrub heath, herb-and cryptogam-dominated meadow, and a *Salix*-dominated shrub community in the low-alpine zone of Central Norway. We measured day-time mid-growing season gross ecosystem photosynthesis (GEP) and ecosystem respiration (ER) together with community vegetation structure represented by biomass of functional groups. We addressed two main research questions: (a) How does herbivore exclusion affect standing biomass and carbon sequestration in heath, meadow, and *Salix* shrub communities? and (b) How does introduction of *Salix* transplants into heath and meadow affect standing biomass and carbon sequestration, and does herbivory affect these changes?

We hypothesize that cessation of grazing and browsing and *Salix* introduction will increase gross ecosystem photosynthesis (GEP) and ecosystem respiration (ER). Even though this has often been suggested in the literature, to our knowledge no other studies have tested this, which makes this study specifically timely.

We further hypothesize that the treatment effects will be greatest in the meadow, as this is the community with the most palatable vegetation.

## Methods

### Study site

The study was performed in the low-alpine vegetation zone around 1100 m a.s.l. in Dovrefjell, Central Norway (62°N, 9°E) (see Additional file 1: Figure S1). The area has a continental climate [58], and from 1960 to 1990 the annual and growing season mean temperatures were − 1 °C and 7.1 °C, respectively, and mean precipitation for the same periods was 700 mm and 298 mm [59]. In 2015, the annual and growing season mean temperatures were 1.58 °C and 8.15 °C, respectively, and the mean precipitation for the same periods was 667 mm and 265 mm at the closest weather station at Hjerkinn, 1012 m a.s.l. (Norwegian Meteorological Institute, eklima.met.no). The study sites were above the forest line, and we put up plots in an *Empetrum*-dominated heath, an herb-and cryptogam-dominated meadow, and a *Salix*-dominated shrub community. The heath was dominated by low-growing dwarf shrubs, and a few graminoids, lichens, and bryophytes. The meadow was more species rich and dominated by graminoids, forbs together with lichens and bryophytes. A few dwarf shrubs and seedless vascular plants were additionally present in the meadow. The shrub community consisted of a deciduous shrub canopy with an understory dominated by graminoids, forbs, and a thick layer of lichens and bryophytes (see detailed plant species in Additional file 1: S1).

The three communities were situated on podzolic soil profiles, with a partial albic horizon in the shrub community and a well-developed albic horizon in the heath [60]. All three communities had a thick layer of till deposits from glacial moraines. Underlying bedrock in the heath and the shrub communities was metavolcanic bedrock, while the meadow community was underlain by shale [61]. The heath and shrub community were south facing, whereas the meadow was south-west facing.

Snow cover during winter in the meadow and shrub community is deep (March 2015 snow depth in the meadow was 38 ± 4.4 cm and in the shrub community it was 51 ± 24 cm), while it is more unstable and often shallow in the heath (March 2015 snow depth was 0 cm) [17]. Animal husbandry in the area began about 400 years BC, and probably intensified around year 700 with permanent settlement [62]. Before 1970, animal husbandry in the study area included horses, cows, and sheep, but after 1970 when most farms specialized in one animal the area was mainly used for domestic Norwegian white sheep (*Ovis aries*) (Vegar Nystuen, personal communication). From the 70 s to the present the number of sheep in the area has been relatively stable (Vegar Nystuen, personal communication) with low-intensity summer grazing and browsing with up to 25 sheep per km^2^ [63]. Voles (*Microtus agrestis*, *M. oeconomus*, and *Myodes rufocanus*) and lemmings (*Lemmus lemmus*) are also present, and the area experienced rodent peak years in 2007, 2011, and 2014 [50]. Other larger herbivores present or passing through the sites are ptarmigan (*Lagopus lagopus* and *L. muta*), hare (*Lepus timidus*), moose (*Alces alces*), and occasionally wild reindeer (*Rangifer tarandus*).

In summer, meadows are important for sheep summer grazing, as they are more productive and nutrient rich than heaths, whereas heaths are common resting sites for the sheep [49, 55, 64]. In early summer, *Salix* twigs in shrub communities are browsed by sheep [65], and *Salix* shrubs may provide both forage and shelter for smaller animals such as ptarmigans and rodents during both summer and winter [52, 66]. During winter, deep snow cover in meadows and shrub communities is important for rodents that are feeding on bryophytes [67], and *Salix* twigs above the snow are browsed by ptarmigan [68]. The heaths are also easily accessible for winter grazing, because of the shallow snow cover [49]. The three different types of plant communities may therefore respond differently to changes in herbivore exclusion [42].

### Experimental design

**Fig. 1:**
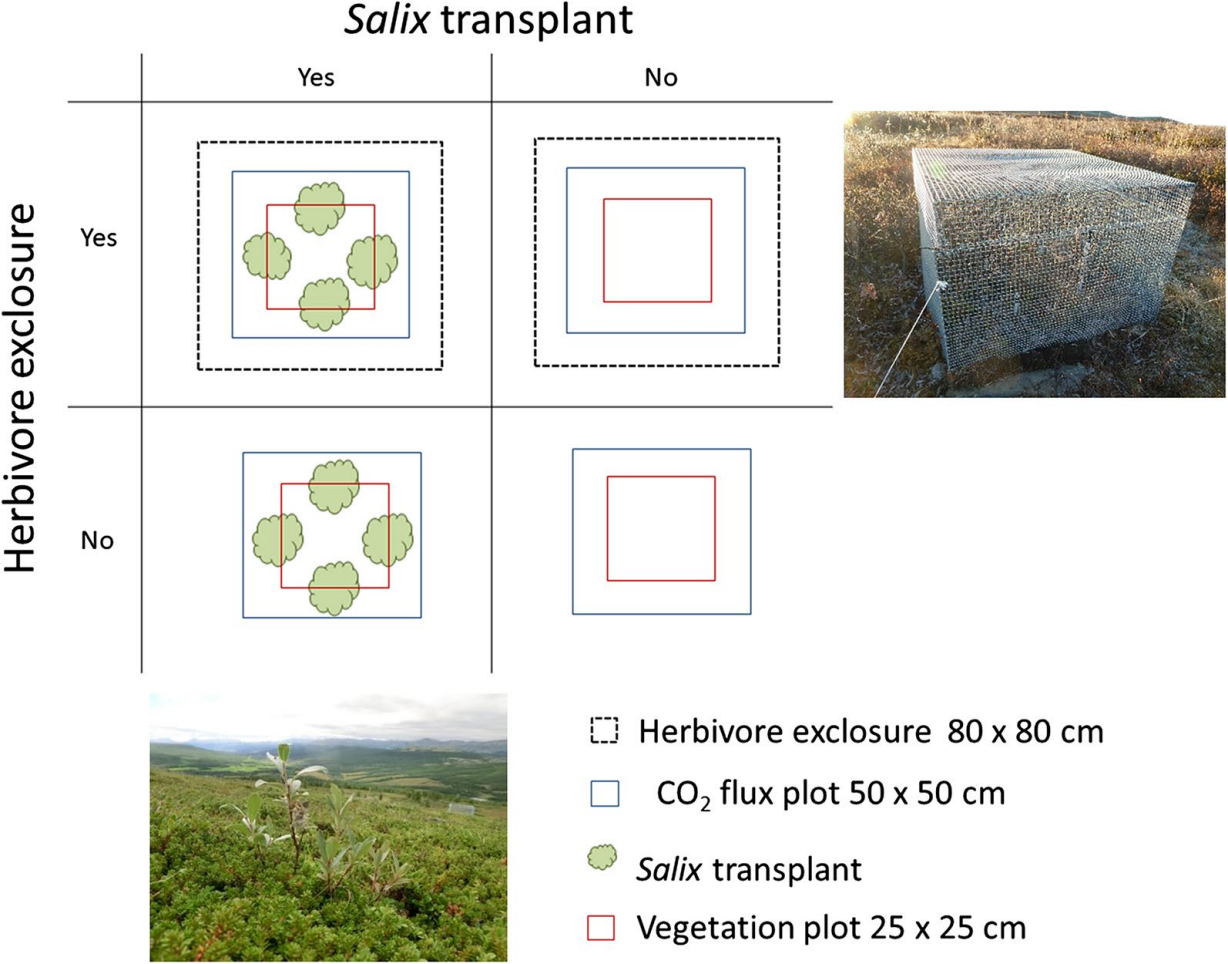
Experimental plot combination of the 2 × 2 factorial design, four treatments replicated eight times in each of three alpine plant communities in the Dovre Mountains, Central Norway. The treatments consisted of plots with and without herbivore exclosures, and plots with and without S*alix* introduction with four *Salix* transplants, that were a mixture of *Salix glauca* and *S. lapponum*. Top right photo is an exclosure in the meadow community and lower left photo is a *Salix* transplant in the heath

In late June 2013, eight blocks were randomly selected for treatment in each community. In each block, we established four plots with different experimental treatments: Plots with and without herbivores and plots with and without *Salix* transplants. The experiment hence was a 2 × 2 factorial design in eight replicates (Fig. 1).

We excluded both small and large herbivores with 80 × 80 × 50 cm exclosures with a lid in early July 2013. The exclosures were made of galvanized steel mesh with mesh size 1.27 × 1.27 cm [12, 69–71], that were dug about 5 to 10 cm into the ground (Fig. 1).

To introduce *Salix*, four *Salix* transplants or rooted willow cuttings, were planted into half of the plots. We used *Salix* plants from a mixed cultivation of *Salix glauca* L. and *S. lapponum* L., and the young plants were not yet possible to distinguish to species. Cuttings of *S. glauca* and *S. lapponum* were collected in the vicinity of the experimental sites in October 2013 and brought to a plant nursery (Norske Naturplanter AS, Færvik, Norge). The cuttings were stored at 0 °C until January 2014, and then planted vertically in commercial plant soil and cultivated during the winter in a greenhouse. In May the willows were planted in 10 cm diameter pots, put outside and topped (long twigs were cut to improve below-ground growth). By June 2014 the plants were trimmed to about 10 cm height, before planting in the field [72]. In 2015, the average height of the plants was 12.6 ± 3.6 SD cm. Mortality was two out of 192 in 2015, both outside the exclosures, one in each the meadow and shrub community. In 2015, a harvest plot was established within each block for biomass measurements (see below).

### Carbon fluxes

Carbon dioxide fluxes were measured in each of the 50 × 50 cm experimental plots (177 measurements on 96 plots, of these 10 plots were measured thrice, 61 plots were measured twice, and 25 plots measured once). All measurements were done on sunny days during mid-growing season in 2015. Fluxes were measured using a closed system composed of a collapsible 0.5 m × 0.5 m × 0.6 m polyethylene chamber and a LI-840A CO_2_/H_2_O infrared gas analyzer (LI-COR Inc., Lincoln, Nebraska, USA), sealed with a 5 m long chain weighing 5 kg. The air inside the chamber was mixed by four fans 30 s prior to and during each measurement. For dark measurements we used an opaque hood to block out the light [73]. Photosynthetically active radiation (PAR) during dark measurements ranged from − 2 to 128 µmol m^−2^ s^−1^, and flow rate averaged 0.8 L min^−1^. To determine the rate of the CO_2_ change in the chamber we first corrected the CO_2_ concentration for water content (C’) and then used linear regression to find the CO_2_ flux (Jasoni and others 2005):

$$C^{\prime} = \frac{{\left[ {CO_{2} } \right]\left( {\upmu{\text{mol}} mol^{ - 1} } \right)}}{{\left[ {H_{2} O} \right]\left( {{\text{mmol}} {\text{mol}}^{ - 1} } \right)}}$$

$$CO_{2}\; flux = \frac{V P }{{R T_{air} \;S}}\frac{{d^{\prime}C}}{dt}$$

where V is the Volume chamber (m^2^), P is the air pressure (kPa) (estimated to be 90 kPa at our sites at 1100 m elevation), R is the the ideal gas constant (8.314 J mol^−1^ K^−1^), T_air_ is the average air temperature (°C) during the measurement, S is the surface area (m^2^), d’C/dt is the the slope of linear regression of C’ on time.

Each measurement started 30 s after sealing, lasted 120 s, and consisted of a light and a dark measurement. net ecosystem exchange (NEE) and ecosystem respiration (ER) were calculated from those measurements respectively. Gross ecosystem photosynthesis (GEP) was calculated by subtracting ER from NEE. When reporting NEE and GEP, negative values denote that the plot is a CO_2_ sink whereas positive values represent a CO_2_ source. To control for variable light intensities during different times of the day, we performed light curve measurements using three levels of shading and standardized GEP to 600 µmol m^−2^ s^−1^ PAR in all control plots and half of the treatment plots (see Additional file 2: S2). However, due to lack of significant differences from non-standardized results, we use non-standardized GEP data in the final results (see Additional file 2: Figure S2).

During all flux measurements, we measured PAR with a LI-190S quantum sensor (LI-COR Inc., Lincoln, Nebraska, USA), air temperature with PT100 sensors inside (at 40 cm height) and outside the chamber (at a height of 60 cm), soil temperature at 8 cm depth, and soil moisture at 5 cm depth with a TRIME-PICO32 sensor (IMKO, Germany). Surface temperature (at 1 cm depth) was interpolated from daily measurements every 4 h with temperature sensors (iButtons, Maxim Integrated Products, Sunnyvale, CA, USA).

For more details on the flux measurement methods see Sørensen et al. [17].

### Vegetation recording and biomass estimation

Vegetation composition was recorded with the point intercept method [74] during the mid-growing season 2015 in the vegetation plot of all the experimental plots (n = 96) with a 25 × 25 cm quadrat and 25 pins (Fig. 1).

To convert the point intercept data into standing biomass for the experimental plots, vegetation was destructively sampled from the harvest plots. Due to logistics, only six out of eight blocks were randomly selected per community (total n = 18). Previous to harvest, vegetation composition was recorded by pin pointing with 25 pins in 25 × 25 cm quadrats in the heath and the meadow communities. In the shrub community we used 25 pins in 50 × 50 cm quadrats to capture the more heterogeneous distribution of woody biomass. The harvested vegetation was sorted into plant functional groups based on growth form, a partitioning that has been shown useful for predicting vegetation effects on ecosystem processes [75]. Prior to harvest, we determined C flux and functional group composition of the harvest plots, and they were not significantly different from the experimental control plots [17]. The functional groups were deciduous shrubs (*Salix glauca*, *S. lapponum*, *Betula nana*), dwarf shrubs (the evergreen shrubs *Empetrum nigrum*, *Vaccinium vitis*-*idaea* and the low growing deciduous shrubs *V. uliginosum*, *V. myrtillus*, *S. herbacea*, and *S. reticulatum*), forbs, graminoids, seedless vascular plants, lichens, and bryophytes. The biomass was oven-dried at 70 °C for 72 h before weighing to an accuracy of 0.001 g. To interpolate from measured biomass to estimated biomass in the experimental plots, the harvested biomass was first converted to g m^−2^ and then regressed on the absolute abundance (number of hits) of each functional group. We followed Jonasson [74] and tested four different regression models for each functional group, and chose the best model based on r^2^ (ranging from 0.564–0.999) and the normal distribution of the model residuals (see Additional file 3: Table S1 and S3). We used parametric bootstrapping with 1000 replicates to get model mean estimates of biomass and 95% confidence intervals. We used the mean estimates in the results. Despite considerable variation in lower and upper limits of confidence intervals, we expect the model means to represent the vegetation biomass well (see Additional file 3: Figure S3). The biomass models were fitted across communities, except for the deciduous shrub, bryophyte, and lichen models (see more Additional file 3: S3).

In the heath community, there was considerable browning of some of the evergreen shrub leaves (*E. nigrum, Arctostaphylus uva*-*ursi, V. vitis*-*idaea)*, which could be due to frost drought damage during the winter [76]. In the biomass models this was assumed to be live biomass, because the branches still seemed alive. There was frost damage on the evergreen shrubs both inside and outside the exclosures, so we assume that this did not affect differences in standing biomass and C fluxes.

### Statistical analysis

We used one-way ANOVA to test for community differences in total biomass and tested significance using multiple comparisons with a Tukey’s honest significant difference test.

As we were interested in the treatment effects *within* each community, the data were analyzed separately for each community. To determine differences in estimated biomass of the functional groups we used factorial 2 × 2 ANOVA with exclosure, transplant, and their interaction as explanatory variables. When data did not meet the assumptions of the parametric analysis, they were ln-transformed. If transformation was not sufficient, we used non-parametric Kruskal–Wallis test (PMCMR package; [77] with one parameter representing all treatment combinations (C—control, E—exclosure, ET—exclosure and transplant and T-transplant). Dunn’s test of multiple comparisons of ranked sums were used to identify treatment differences (dunn.test package; [78]).

To estimate the differences in means of C fluxes between the treatments we used linear mixed effects models following Gaussian distributions (lme4 package; [79]). Gross ecosystem photosynthesis (GEP), net ecosystem exchange (NEE), and ecosystem respiration (ER) were ln-transformed to meet model assumptions. Within the flux models, fixed effects were exclosure, transplant, and their interaction. To control for repeated measurement during season, plot was considered a random effect. Community was additionally a fixed effect, when comparing communities. To evaluate importance of community and treatments for GEP, ER, and NEE, we used model selection with AICc (dAICc < 2) as selection criteria on linear mixed effects models with all interactions (MuMIn package; [80]). Treatment differences in GEP, NEE, and ER among the communities were compared using linear mixed effects models with the Tukey method (multicom package; [81]) on full models without interactions. R Core Team [82] was used for all data analysis.

## Results

### Standing biomass and carbon sequestration in the three plant communities

**Fig. 2:**
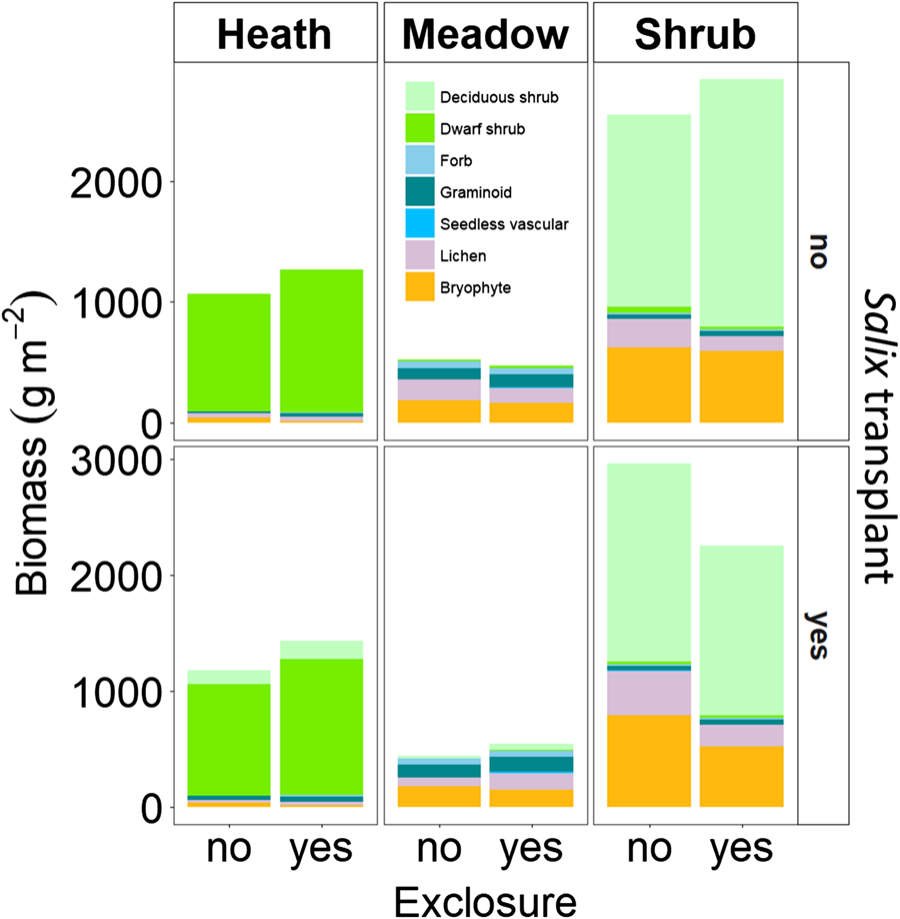
Mean standing biomass of functional groups in plots with and without herbivore exclosure and plots with and without *Salix* transplants. The biomass was estimated from vegetation analysis and harvest performed on harvest plots during mid-growing season for alpine *Empetrum*-heath, meadow and *Salix*-shrub plant communities in the Dovre Mountains, Central Norway. We used parametric bootstrapping with 1000 replicates to get model estimates of biomass. Model performance and estimates with 95% confidence intervals are available in Additional file 3: Table S1 and Figure S3 respectively

**Fig. 3:**
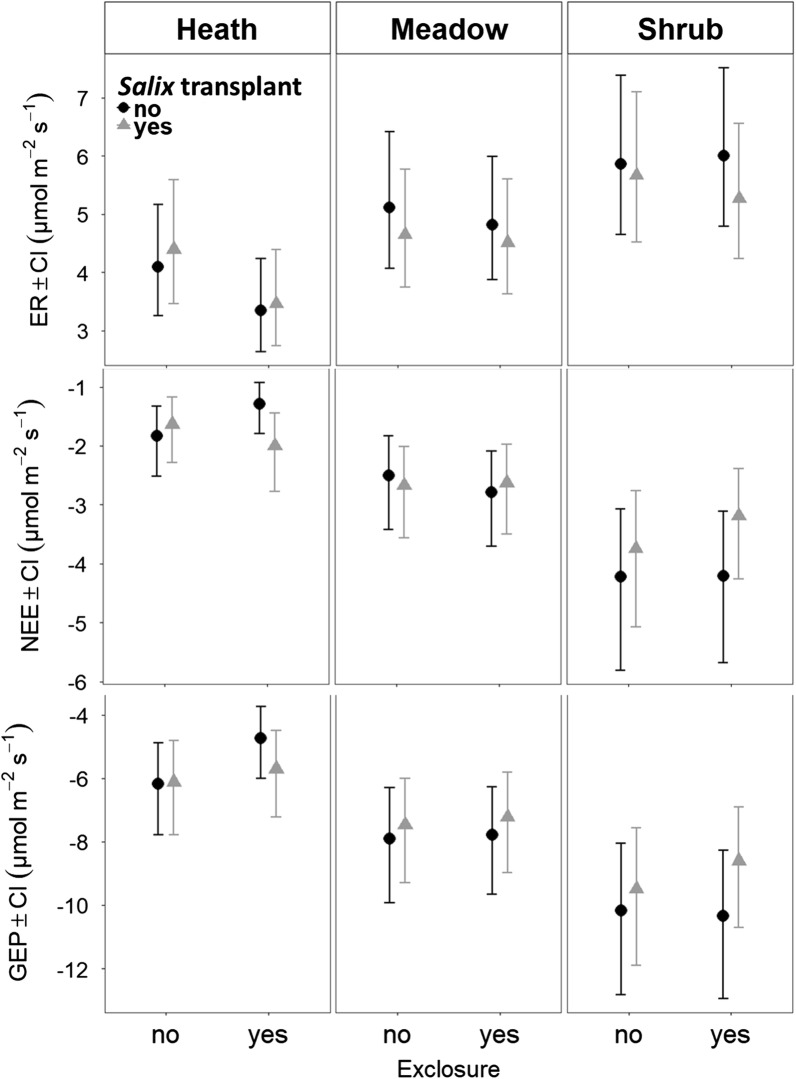
Mean CO_2_ flux estimates (µmol m^−2^ s^−1^) ± 96% confidence interval (CI) on plots with and without herbivore exclosures combined with and without *Salix* transplants during mid-growing season for alpine *Empetrum*-heath, meadow and *Salix*-shrub plant communities in the Dovre Mountains, Central Norway. Estimates based on linear mixed models with plot as a random factor (n = 177). Top: Ecosystem respiration (ER). Middle: Net ecosystem exchange (NEE). Bottom: gross ecosystem photosynthesis (GEP). Within the heath ER was significantly lower with exclosure (*p* = 0.021, Tukey)

As expected, the meadow, heath and *Salix* shrub communities were significantly different with respect to both community structure and carbon sequestration. The shrub community had highest standing biomass followed by heath whereas it was smallest in the meadow community (*p* < 0.0001 for all differences, TukeyHSD) (Fig. 2). Carbon (C) sequestration was greatest in the shrub community (*p* < 0.01, Tukey) with gross ecosystem photosynthesis (GEP) ranging from − 9.54 to − 11.77 µmol m^−2^ s^−1^ and Net Ecosystem Exchange (NEE) ranging from − 3.76 to − 4.79 µmol m^−2^ s^−1^ (Fig. 3). The meadow community had larger GEP (− 7.35 to − 8.10 µmol m^−2^ s^−1^) and NEE (− 2.71 to − 3.01 µmol m^−2^ s^−1^) than the heath (GEP: − 5.27 to − 6.46 µmol m^−2^ s^−1^, NEE: − 1.65 to − 2.25, and *p* < 0.001, Tukey). Ecosystem Respiration (ER) was higher (*p* < 0.01, Tukey) in both the shrub (5.77 to 6.78 µmol m^−2^ s^−1^) and meadow communities (4.64 to 5.26 µmol m^−2^ s^−1^) than in the heath community (3.60 to 4.50 µmol m^−2^ s^−1^) (Fig. 3).

The best models for all the three C fluxes (GEP, NEE, ER) contained only community (see Additional file 4: Table S2).

### Effects of herbivore exclosures

In the heath community herbivore exclusion resulted in increased standing biomass (F_1, 28_ = 5.28, *p* < 0.05, ANOVA), due to increased biomass of dwarf shrubs (F_1, 28_ = 4.52, *p* < 0.05, ANOVA) (Fig. 2). Within exclosures in the heath, there was a tendency for increased biomass of graminoids (*p* = 0.0897, Kruskal–Wallis) (Fig. 2). The biomass of bryophytes was very low in the heath, and even lower within the exclosures (*p* < 0.05, Dunn’s test). In the heath, there was marginally less GEP with herbivore exclosures (*p* = 0.082, Tukey) and ER was significantly reduced (*p* < 0.05, Tukey) (Fig. 3).

Within the meadow and shrub communities there were no significant effects of herbivore exclusion on biomass or carbon fluxes. However, the shrub community was significantly (*p* < 0.05) drier and cooler inside than outside the exclosures during C flux measurements. In the meadow community surface temperature was significantly (*p* < 0.05) lower inside the exclosures than outside (Additional file 5: TableS3). This corresponds to generally cooler surface temperatures inside the exclosures during summer in both the heath and meadow communities. In the meadow community, surface temperatures were additionally warmer during winter. At the time of snow depth measurements, there was about one cm snow within exclosures in the heath, and this was significantly more than outside the exclosures where there were none. Otherwise there were no significant differences in snow depth in any of the other communities (Additional file 5: Table S4). Thus, the effect of reduced grazing is difficult to disentangle from other exclosure effects.

### Effects of *Salix* transplantation

In the heath, transplantation of *Salix* not only increased the biomass of deciduous shrubs (F_1, 28_ = 18.84, *p* < 0.001, ANOVA) but also that of graminoids (*p* < 0.05, Dunn’s test). There was also an increase in biomass of deciduous shrubs due to the transplantation of *Salix* in the meadow but surprisingly, it was not significant (F_1, 28_ = 3.18, *p* = 0.086, ANOVA) (Fig. 2). The transplantation of *Salix* did not affect the C fluxes significantly in any of the communities.

### Treatment interactions

Herbivore exclusion did not show significant interactions with transplantation in the heath and the meadow (Figs. 2 and 3). In the shrub community, there was higher standing biomass (F_1, 28_ = 5.74, *p* < 0.05), with exclosure and no transplant than with both exclosure and transplant (Fig. 2).

## Discussion

The aim of this study was to evaluate short-term effects of *Salix* shrub expansion and herbivory on community structure and carbon (C) cycling in arctic-alpine tundra. Contrary to expectations, the effects of the treatments were strongest in the heath. After just 2 years, exclusion of herbivory increased the standing biomass in the heath due to increased biomass of dwarf shrubs. The biomass of bryophytes was reduced as was the ecosystem respiration (ER). *Salix* introduction effects were weak, despite increased biomass of graminoids and deciduous shrubs in the heath community. There were no treatment effects on the standing biomass or C fluxes in the two other community types.

### Effects of herbivore exclusion

In this study, we excluded both small and large herbivores, and previous studies have reported significant results after only two growing seasons [12, 69, 71]. However, the increased standing biomass in the heath and not in the meadow community were unexpected since evergreen dwarf shrubs (which mainly contributed to the increased standing biomass) are often avoided by herbivores due to their high content of secondary compounds [83–85]. However, the increase was caused by increased growth of the evergreen shrubs, similar to previous exclosure studies in tundra [14, 37, 86]. It was not an artefact of the categorization of *Betula nana* as a dwarf shrub in the heath biomass models, since an analysis of dwarf shrub abundance without *B. nana* showed similar results (*χ*^2^ (1) = 5.27, *p* < 0.05, n = 31, Likelihood ratio test).

The entire study area has low-intensity sheep grazing, but lack of trampling from the sheep may have caused the effect of increased standing biomass with exclusion of herbivores [84, 87]. Sheep often tend to rest and chew their cud in lichen heaths and similar dry, low-statured vegetation [49]. An installation with salt licks attracting sheep is located about 500 m from the heath site, and we observed sheep feces, torn out plot marking poles, and wool on the exclosures. The sheep most likely graze on the forbs and to some extent the graminoids, and this could explain the marginally higher graminoid biomass with cessation of grazing. Moreover, sheep grazing has been shown to favor the resistant *Polytrichum* species that are present in the heath community [88] and this could contribute to explaining the reduced bryophyte biomass in the exclosures. Wild reindeer occasionally pass through the area, and their presence may have added to the reduced standing biomass outside exclosures in the heath. However, reindeer prefer lichen and since there is low abundance of lichen in the heath community, this is less likely. Still, reindeer winter grazing due to the shallow snow cover in the heath community could have played a role, even though dwarf shrubs are not of high preference to reindeer [85]. Despite a rodent peak in 2014, rodent densities during the study years in the study area were not very high [50], so we believe that the influence of rodents was minimal. Moreover, rodent grazing is most pronounced in the winter and they prefer a deep snow cover over the shallow cover found in the heath [67].

The decrease in bryophyte abundance when vascular plant biomass increases after excluding herbivores or warming with open top chambers has previously been shown in similar vegetation [89–92]. This could be associated with decreased light levels due to shading by the vascular plants [91], though it could also be an exclosure effect. In the meadow, the exclosure with lid resulted in a difference in light intensity (PAR) ranging from 11 to 29% depending on whether it was sunny or cloudy (Nystuen, unpublished) and we assume that there would be similar differences in the heath and shrub communities. The reduced light could have affected the treatments. The microclimate was generally cooler inside the exclosures during summer in the heath and the meadow. During winter, surface temperatures were warmer in exclosures in the meadow, even though snow depth was significantly different only within the heath. Thus, the effect of reduced grazing or reduced trampling is difficult to distinguish from other exclosure effects. In previous studies using exclosures with a small mesh size and lid [12], lack of continuous microclimatic surveys combined with high herbivory levels could have disguised such a potential exclosure effect. These side effects parallel problems of using open top chambers [93], and we need to find a way to eliminate this problem. In the present study, the positive treatment effect of herbivore exclusion could have been negated by a negative treatment impact of the exclosure itself. Alternatively, the lack of effects of herbivore exclusion on the standing biomass in the meadow and shrub communities suggest low herbivory levels during the experiment in these communities. We expected the effect of rodent grazing to be most pronounced in the meadow and shrub communities, but the few rodents during the years of the experiment could explain why we did not see any treatment effects in these communities.

Related to the small treatment effects on the standing biomass we also saw few effects on the fluxes. However, the significantly lower ER in exclosures in the heath was unexpected given the greater standing biomass. A similar trend was found with sheep grazing in temperate grasslands [38]. A possible mechanism for reduced ER in the heath could be reduced N-input within the exclosures: In the area with sheep present, feces and urine might enhance N input and increase N mineralization and microbial activity [87], which in turn could increase C cycling through increased heterotrophic respiration. We found no evidence of exclosure shading effects on the flux measurements. In the heath community where we found decreased ER with exclosures, there were no difference in surface temperature during the flux measurements (Additional file 5: Table S3). The only effects of exclosures on microclimate during the flux measurements, was lower surface temperature in the meadow, and drier soil and cooler air temperature in the shrub community. This was surprising, but the cooler temperatures may have been caused by shading from the sides of the cages, whereas the drier soils in the shrub community may indicate higher evapotranspiration in the plots and perhaps an undetected increase in *Salix* canopy density.

### Effects of shrub expansion in heath and meadow communities

Our use of *Salix* introduction in the present study provides a method to experimentally test effects of shrub expansion, whereas previous studies have used succession or natural *Salix* recruits [13]. This experiment will bring interesting data in the years to come, as this method provides knowledge of the exact successional history at plot-scale. In 1 year and almost two growing seasons, the *Salix* transplants in our experiment had not reached a size where they affected the ecosystem substantially, but these results provide important baseline data for future analysis. Still, graminoid biomass was significantly greater with *Salix* introduction in the heath community. However, due to the low overall graminoid biomass in this community, the increased graminoid biomass was not reflected by the C flux measurements.

The lack of shrub introduction effects in the meadow and heath community is not that surprising because community processes in arctic-alpine tundra often are very slow. The results of our study may indeed confirm that *Salix* establishment in closed vegetation is very slow compared to establishment on bare soil [57], and potentially growing them bigger and taller before planting them out and taking mycorrhizal symbionts into account could be an idea for future study designs [94].

### Treatment interactions—experimental manipulations?

The *Salix* introduction in the shrub community was done as a control, since we did not expect to find any differences in this community. The significant interaction in standing biomass with herbivore exclosures and *Salix* transplants in the shrub community was therefore puzzling. This might be an artefact of the heterogeneous nature of the plots in this community. Another possibility is that these plots have the most disturbed shrub canopies, since we both have manipulated the vegetation by digging to establish the exclosures and planting the *Salix* transplants. This may unfortunately have systematically affected the standing biomass in those plots, at least in the short term.

## Conclusion

Our short-term study demonstrated that both shrub introduction and herbivore exclusion had small but still significant effects on alpine tundra heath vegetation, whereas such effects were not apparent in the herb-and cryptogam-dominated meadow and the *Salix*-dominated shrub community. We demonstrated that there is a potential exclosure side effect altering the microclimate. We found a significant increase in above-ground biomass in the heath with herbivore exclosures. This could be an effect from reduced trampling, but can also be a shading effect from the exclosure. Following the treatments over more years is needed to estimate the long-term effects on community structure and the consequences for carbon sequestration in the three plant communities. Such data are important for predicting the impact of shrub expansion on C budgets from alpine regions.

## Additional files

**Additional file 1: Figure S1.** Location of study site.** S1. **Detailed description of community plant species.

**Additional file 2: S2.** Method for standardization of Gross Ecosystem Photosynthesis (GEP) to 600 PAR. **Figure S2.** Comparison of GEP to GEP600.

**Additional file 3: S3.** Biomass models method. **Table S1.** Model performance of linear models used to estimate biomass in experimental plots. **Figure S3.** Mean biomass model estimates with upper and lower 95 % confidence interval. **Figure S4.** A stacked barplot of mean standing biomass in experimental plots illustrating implications of dwarf shrubs categorization.

**Additional file 4: Table S2.** Carbon flux model selection.

**Additional file 5: Table S3.** Mean environmental variables during CO_2_ flux measurements. **Table S4.** Mean summer and winter surface temperature and snow depth.

## Abbreviations

a.s.l.: above sea level
AIC: Akaike information criterion
AICc: Akaike information criterion corrected for small sample size
B: harvest plot
BC: before Christ
C: carbon
C: one of four treatments, control
C’: CO_2_ concentration corrected for water content
CI: confidence interval
CO_2_: carbon dioxide
d’C/dt: the slope of linear regression of C’ on time
dAICc: delta AICc, AICc difference from the top model
df: degrees of freedom
E: one of four treatments, exclosure treatment
ER: ecosystem respiration
ET: one of four treatments, exclosure and transplant treatment
GEP: gross ecosystem photosynthesis
GEP600: GEP standardized to 600 PAR
I: incident PAR (photosynthetic active radiation)
k: half saturated constant of photosynthesis
Moisture: soil moisture
N: nitrogen
n: number of observations
NEE: net ecosystem exchange
P: air pressure
PAR: photosynthetic active radiation
P_max_: rate of light saturated photosynthesis
Q10: the ratio of rates given a 10 C change in temperature
R: the ideal gas constant
R conditional: the proportion of variance explained by both fixed and random factors
R marginal: the proportion of variance explained by fixed factors
S: surface area
SD: standard deviation
SE: standard error
T: one of four treatments, transplant treatment
T_air_: average air temperature inside chamber
T_soil_: soil temperature at 8 cm depth
T_surface_: temperature at 1 cm depth
V: volume chamber
*χ*^2^: chi square
