# Supplementary material for: Experimental herbivore exclusion, shrub introduction, and carbon sequestration in alpine plant communities
Source: BMC Ecol. 2018 Aug 30;18:29. doi: 10.1186/s12898-018-0185-9 (PMC6117883; doi:10.1186/s12898-018-0185-9)
Supplement: Supplementary file 1 — Additional file 1: Figure S1. Location of study site. S1. Detailed description of community plant species. [file 12898_2018_185_MOESM1_ESM.pdf]

Additional file 1
For
Experimental herbivore exclusion, shrub introduction, and carbon sequestration in alpine plant communities

Author names: Mia Vedel Sørensen*, Bente Jessen Graae, Dagmar Hagen, Brian J. Enquist, Kristin Odden Nystuen, Richard Strimbeck

***Corresponding author**: Mia Vedel Sørensen, miavedelsorensen@gmail.com

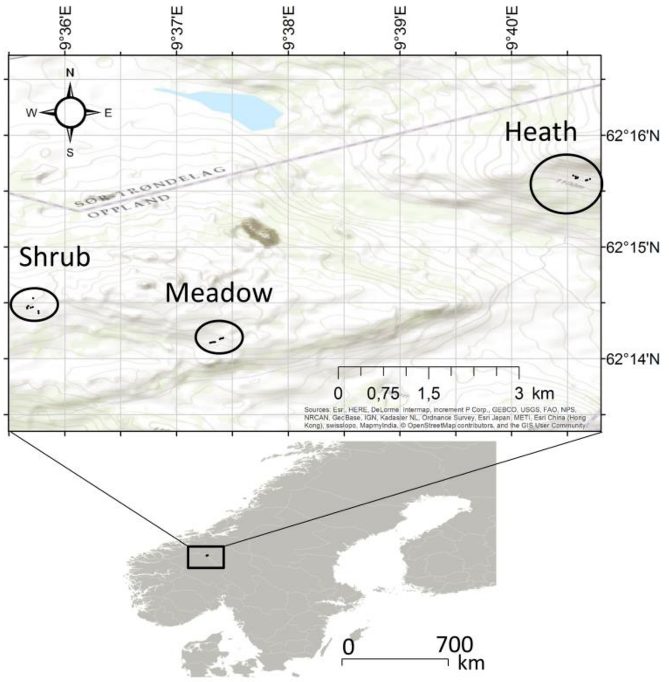

Figure S1: Location of study site and the three plant communities, in Dovre Mountains, Central Norway.

S1: Detailed description of community plant species

The heath was dominated by low growing dwarf shrubs (e.g., *Empetrum nigrum hermaphroditum*, *Arctostaphylos uva-ursi*, *Vaccinium vitis-idaea*, *V. uliginosum*, *Loiseleuria procumbens*, *Betula nana*), a few graminoids, especially *Festuca ovina*, and lichens (e.g., *Flavocetraria cucullata*, *Cladonia mitis*, *C. rangiferina*, *C.* s.l *gracilis*, *Alectoria ochroleuca, Cetraria islandica*) and bryophytes (e.g., *Polytrichum commune*, *P. juniperinum*, *Dicranum flexicaule*, *Ceratodon purpureus*) were present.

The meadow was more species rich and dominated by graminoids (e.g., *Avenella flexuosa*, *Festuca ovina*, *Anthoxanthum nipponica*, *Deschampsia cespitosa*, *Carex bigelowii*, *C. vaginata*, *Juncus trifidus, Agrostis capillaris*), forbs (e.g., *Thalictrum alpinum*, *Antennaria dioica*, *Leontodon autumnalis*, *Saussurea alpina*, *Campanula rotundifolia, Astragalus alpinus*, *Ranunculus acris*, *Potentilla crantzii*, *Galium boreale*, *Sibbaldia procumbens*, *Achillea millefolium*, *Bistorta vivipara*, *Cerastium alpinum*) together with lichens (e.g., *Stereocaulon alpinum*, *Cetraria islandica*) and bryophytes (e.g., *Hylocomium splendens*, *Barbilophozia lycopodioides*, *Aulacomnium palustre*, *Hylocomiastrum pyrenaicum*, *Sanionia uncinata*, *Pleurozium schreberi*). A few dwarf shrubs (*Salix herbacea*) and seedless vascular plants (*Selaginella selaginoides*) were present.

The shrub community consisted of a deciduous shrub canopy (*Salix glauca, S. lapponum* and some *Betula nana*) with an understory dominated by graminoids (e.g., *Avenella flexuosa*, *Festuca ovina*, *Carex bigelowii, C. vaginata, Anthoxanthum nipponica*, *Agrostis mertensii*), forbs (*Solidago virgurea*, *Saussurea alpina*, *Thalictrum alpinum*, *Pedicularis lapponica*, *Galium boreale*, *Pyrola minor*, *Trientalis europaea*, *Ranunculus acris*), and a thick layer of lichens (*Cladonia mitis*, *C. stellaris*, *C. Stygia*, *C. rangiferina*) and bryophytes (*Pleurozium schreberi*, *Polytrichum commune*, *Hylocomium splendens)*. A few dwarf shrubs (e.g., *Vaccinium vitis-idaea* L. and *V. myrtillus*) were present.

Nomenclature followed Lid and Lid [1] for vascular plants, Hill et al. [2] for bryophytes, and Artsdatabanken [3] for lichens.

References

1. Lid J, Lid D: **Norsk flora**, 7 edn: Det Norske Samlaget; 2007.
2. Hill MO, Bell N, Bruggeman-Nannenga MA, Brugués M, Cano MJ, Enroth J, Flatberg KI, Frahm JP, Gallego MT, Garilleti R *et al*: **An annotated checklist of the mosses of Europe and Macaronesia**. *Journal of Bryology* 2006, **28**(3):198-267.
3. **Artsnavnebasen. Norsk taksonomisk database.** [http://www.artsportalen.artsdatabanken.no/]
