# Supplementary material for: Experimental herbivore exclusion, shrub introduction, and carbon sequestration in alpine plant communities
Source: BMC Ecol. 2018 Aug 30;18:29. doi: 10.1186/s12898-018-0185-9 (PMC6117883; doi:10.1186/s12898-018-0185-9)
Supplement: Supplementary file 2 — Additional file 2: S2. Method for standardization of Gross Ecosystem Photosynthesis (GEP) to 600 PAR. Figure S2. Comparison of GEP to GEP600. [file 12898_2018_185_MOESM2_ESM.pdf]

**Additional file 2**
**For**
Experimental herbivore exclusion, shrub introduction, and carbon sequestration in alpine plant communities

**Author names:** Mia Vedel Sørensen*, Bente Jessen Graae, Dagmar Hagen, Brian J. Enquist, Kristin Odden Nystuen, Richard Strimbeck

***Corresponding author**: Mia Vedel Sørensen, email: miavedelsorensen@gmail.com

**S2: Method GEP standardization to 600 PAR**

Light curve measurements was performed one time during mid-growing season 2015 on all control plots and half of the blocks in each community with all experimental plots (n = 60). One light curve measurement consisted of one measurement in full light, one measurement at three increasing levels of shading, and one measurement in full darkness [1, 2]. The shading was done with three layers of black tulle.

Light response curves were derived using the nls functions in R [3] with the following equation:

$$GEP = \frac{P_{max} \cdot I}{k + I}$$

Where GEP = ER – NEE (ER corresponding to the dark measurements, and NEE corresponding to the four light measurements), I = incident PAR (µmol $m^{-2}s^{-1}$), $P_{max}$ = rate of light saturated photosynthesis, and k = half saturated constant of photosynthesis.

If we did not have any saturation from the light response curve, we used a fixed value of $P_{max}$, and tried different values, and chose the one with the best fit.

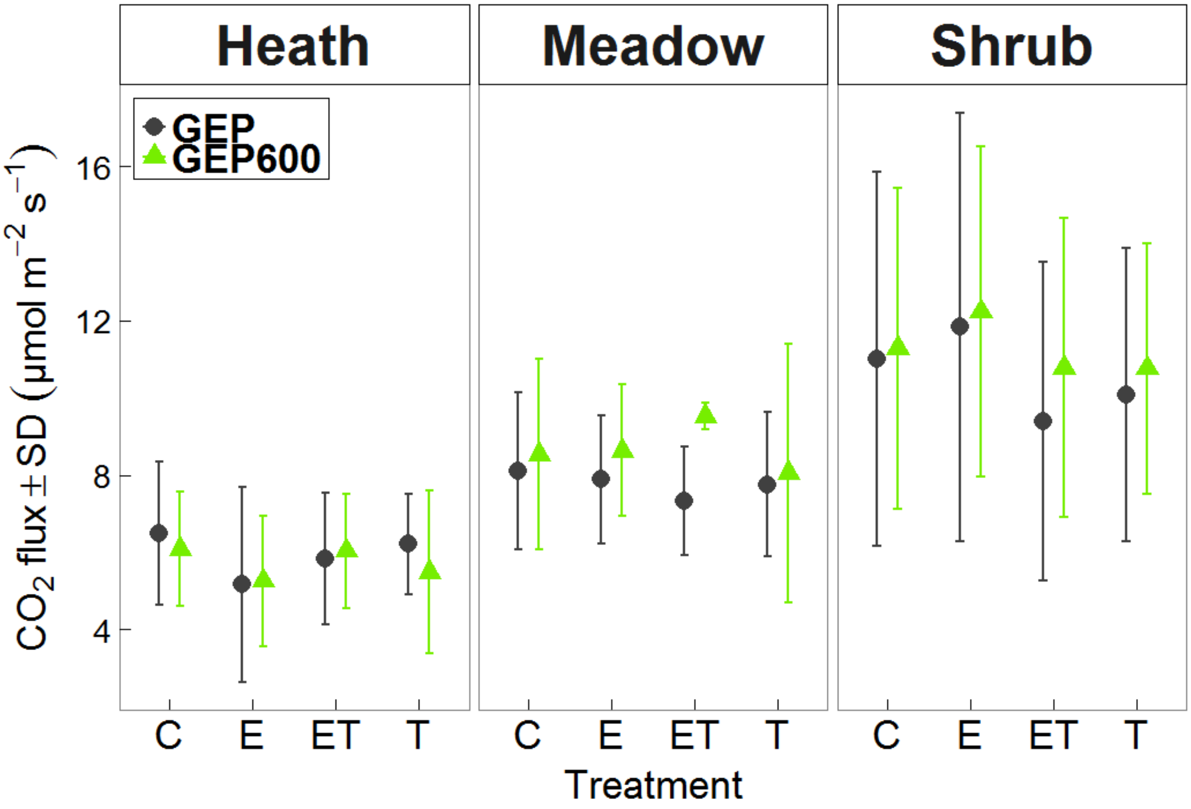


**Figure S2: GEP compared to GEP600.** Differences between Gross Ecosystem Photosynthesis (GEP) and GEP standardized to 600 PAR (GEP600) (μmol m$^{-2}$ s$^{-1}$) for the four treatments control (C), herbivore exclosure (E), *Salix* transplant (T) and exclosure combined with *Salix* transplant (ET) in alpine *Empetrum*-heath, meadow and *Salix*-shrub communities in Dovre Mountains, Central Norway. Differences were tested with one-way ANOVA and there was no significant difference between means of GEP and GEP600 ($F_{1, 22}$ = 1.3, $p$ = 0.621).

## References


1. Williams M, Street. LE., van Wijk M, Shaver G: **Identifying differences in carbon exchange among arctic ecosystem types**. *Ecosystems* 2006, **9**(2):288-304.
2. Street L, Shaver G, Williams M, van Wijk M: **What is the relationship between changes in canopy leaf area and changes in photosynthetic CO2 flux in arctic ecosystems?** *Journal of Ecology* 2007, **95**:139-150.
3. **A language and environment for statistical computing** [https://www.R-project.org/]
