# Supplementary material for: Experimental herbivore exclusion, shrub introduction, and carbon sequestration in alpine plant communities
Source: BMC Ecol. 2018 Aug 30;18:29. doi: 10.1186/s12898-018-0185-9 (PMC6117883; doi:10.1186/s12898-018-0185-9)
Supplement: Supplementary file 3 — Additional file 3: S3. Biomass models method. Table S1. Model performance of linear models used to estimate biomass in experimental plots. Figure S3. Mean biomass model estimates with upper and lower 95 % confidence interval. Figure S4. A stacked barplot of mean standing biomass in experimental plots illustrating implications of dwarf shrubs categorization. [file 12898_2018_185_MOESM3_ESM.pdf]

Additional file 3

For

Experimental herbivore exclusion, shrub introduction, and carbon sequestration in alpine plant communities

Author names: Mia Vedel Sørensen*, Bente Jessen Graae, Dagmar Hagen, Brian J. Enquist, Kristin Odden Nystuen, Richard Strimbeck

*__Corresponding author__: Mia Vedel Sørensen, email: miavedelsorensen@gmail.com

S3: Biomass models method

For each functional group we ran models across community for the harvest plots, to get a relationship between absolute abundance (number of hits) and biomass per each functional group.
Biomass was converted to g m^2, because harvest plots in the heath and meadow was 25 cm x 25 cm and 50 cm x 50 cm in the shrub community.
We followed S Jonasson [1] and tested four different linear models for each functional group. We then chose the best model based on r^2 and the normal distribution of model residuals:

M1: Biomass per functional group ~ absolute abundance functional group
M2: Biomass per functional group ~ ln (absolute abundance functional group)
M3: ln (biomass per functional group) ~ (absolute abundance functional group)
M4: ln (biomass per functional group) ~ ln (absolute abundance functional group)

To get model estimates of biomass and 95% confidence intervals, we used parametric bootstrapping with 1000 replicates.

Deciduous shrub models were based on deciduous shrubs present in the *Salix* shrub community only, because there were no hits of deciduous shrubs in the harvest plots in the other communities.
In the first models, estimated biomass of deciduous shrubs in the heath were therefore unrealistically high in those few plots where some creeping *Betula nana* was present. This was due to the difference in vegetation height between the heath and shrub communities. In the final models, *B. nana* in the heath was therefore categorized as a dwarf shrub, to get more realistic biomass estimates (see figure S4).

Bryophyte and lichen models were run separately for each community. Only the first hit for those two functional groups was counted (i.e., a max. of 25 hits per plot), and as the cryptogam layer was much thicker in the *Salix* shrub community and 24 hits in the meadow and the *Salix* shrub community would cover very different amounts of biomass, we chose to run those models separately for each community. The only exception was the bryophyte model for the heath and meadow communities. This was a joint model, due to only two harvest plots in the heath with bryophytes present, and biomass and hits were more similar in those two communities.

References

1. Jonasson S: **Evaluation of the Point Intercept Method for the Estimation of Plant Biomass**. *Oikos* 1988, **52**(1):101-106.

Table S1: Model performance of linear models used to estimate biomass in experimental plots: r^2, adjusted r^2 (r^2 adj.), Akaike information criterion (AIC), F-statistics, and degrees of freedom (df). Models were based on absolute abundance and biomass in harvest plots of each functional group.

**Functional group**	**r^2**	**r^2 adj.**	**AIC**	**F-statistic**	**df_{num}**	**df_{den}**
Deciduous shrub	0.564	0.455	4	5.17	1	4
Dwarf shrub	0.954	0.951	201	313.26	1	15
Forb	0.933	0.926	12	139.52	1	10
Graminoid	0.954	0.951	18	333.35	1	16
Bryophyte in heath and meadow	0.721	0.674	86	15.50	1	6
Bryophyte in shrub	0.844	0.806	13	21.71	1	4
Lichen in heath	0.695	0.618	53	9.11	1	4
Lichen in meadow	0.804	0.755	63	16.39	1	4
Lichen in shrub	0.962	0.953	8	101.94	1	4
Seedless vascular	0.999	0.999	35	4302.50	1	4

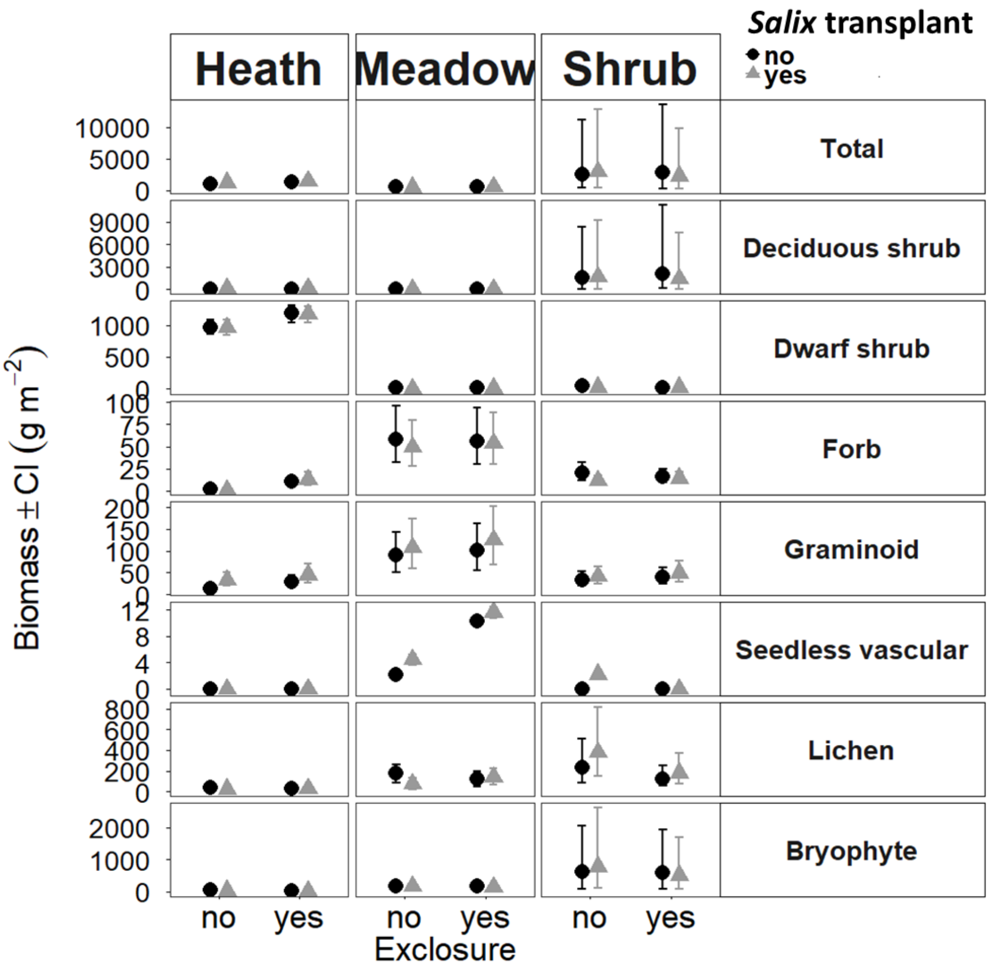

Figure S3: Mean biomass model estimates with upper and lower 95 % confidence interval (CI) for total biomass, and all the functional groups in alpine *Empetrum*-heath, meadow and *Salix*-shrub communities in the Dovre Mountains, Central Norway. Only the mean estimates were used in the standing biomass results in figure 2.

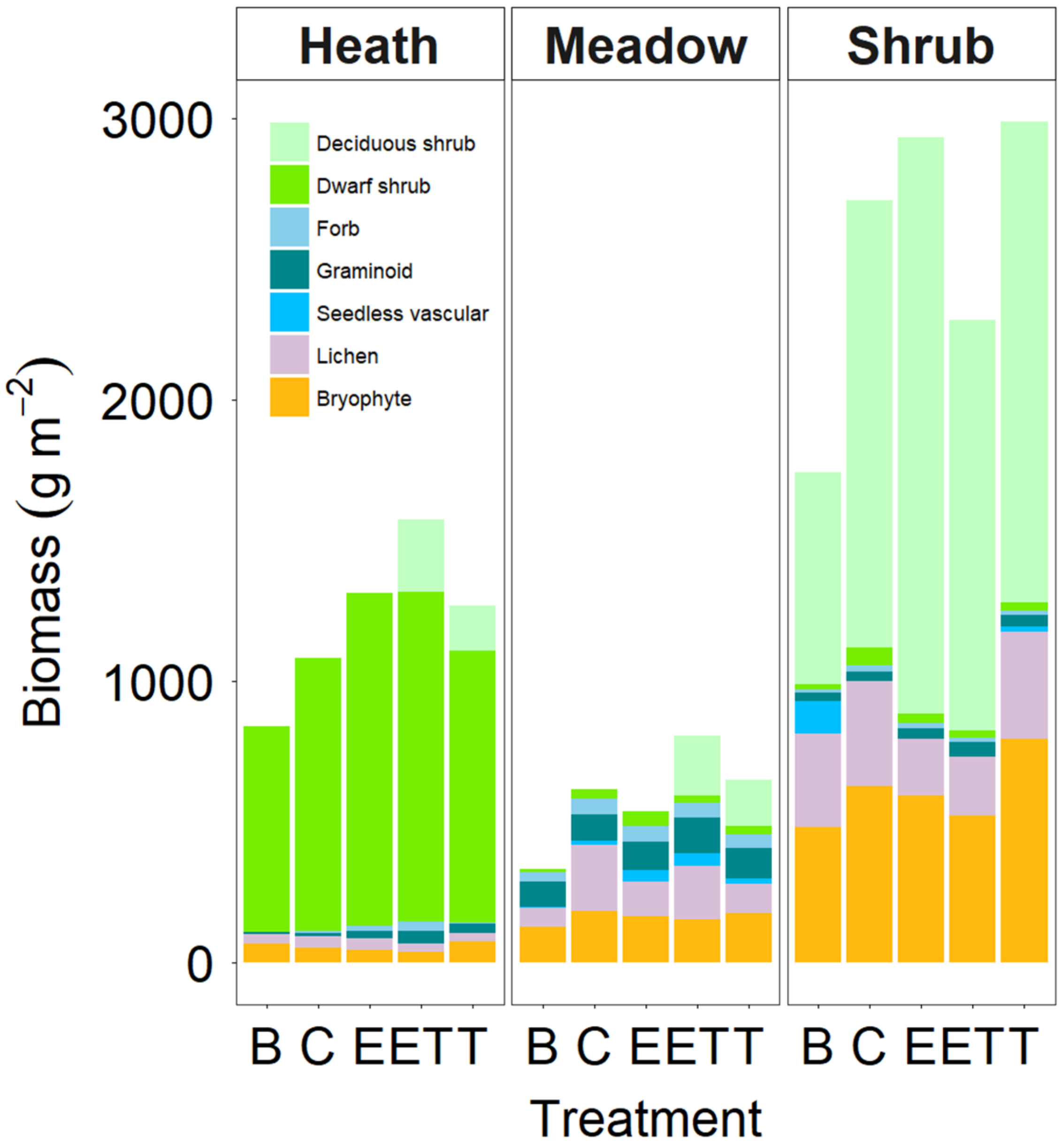

Figure S4: Standing biomass in experimental plots (control (C), herbivore exclosure (E), *Salix* transplant (T), and exclosure combined with *Salix* transplant (ET)) compared to harvest plots (B). Here, deciduous shrubs include *Betula nana*, also in the heath. In the final models, *B. nana* was categorized as a dwarf shrub in the heath, to get more realistic biomass estimates in this community. See explanation above in S3.
