# Supplementary material for: Experimental herbivore exclusion, shrub introduction, and carbon sequestration in alpine plant communities
Source: BMC Ecol. 2018 Aug 30;18:29. doi: 10.1186/s12898-018-0185-9 (PMC6117883; doi:10.1186/s12898-018-0185-9)
Supplement: Supplementary file 4 — Additional file 4: Table S2. Carbon flux model selection. [file 12898_2018_185_MOESM4_ESM.pdf]

Additional file 4

For

Experimental herbivore exclusion, shrub introduction, and carbon sequestration in alpine plant communities

Author names: Mia Vedel Sørensen*, Bente Jessen Graae, Dagmar Hagen, Brian J. Enquist, Kristin Odden Nystuen, Richard Strimbeck

***Corresponding author**: Mia Vedel Sørensen, email: miavedelsorensen@gmail.com

Table S2: **Carbon flux model selection**. Top models and full model without interactions for linear mixed effect models with CO_2 fluxes as response variables (ER=Ecosystem Respiration, NEE=Net Ecosystem Exchange, GEP=Gross ecosystem Photosynthesis), plot as random effects, and community, exclosure, transplant and all interactions as fixed factors. Number of observations (n), the proportion of variance explained by fixed factors (Rmarginal), the proportion of variance explained by both fixed and random factors (Rconditional) [1], Akaike information criterion corrected for small sample size (AICc) and delta AIC (dAICc). According to AICc the models with only community were best.

Measurement	Fixed factors	n	R Marginal	R Conditional	AICc	dAICc
ER	Community	177	0.16	0.57	137	0
ER	Community + Exclosure	177	0.17	0.57	141	4
ER	Community + Transplant	177	0.16	0.57	143	5
ER	Community + Transplant + Exclosure	177	0.17	0.57	147	9
NEE	Community	177	0.27	0.45	290	0
NEE	Community + Exclosure	177	0.27	0.45	295	5
NEE	Community + Exclosure + Transplant	177	0.27	0.46	300	10
GEP	Community	177	0.24	0.58	147	0
GEP	Community + Exclosure	177	0.25	0.58	152	4
GEP	Community + Transplant	177	0.24	0.58	153	5
GEP	Community + Exclosure + Transplant	177	0.25	0.58	157	10

1. Nakagawa S, Schielzeth H: **A general and simple method for obtaining R2 from generalized linear mixed-effects models**. *Methods in Ecology and Evolution* 2013, **4**(2):133-142.
