# Supplementary material for: Experimental herbivore exclusion, shrub introduction, and carbon sequestration in alpine plant communities
Source: BMC Ecol. 2018 Aug 30;18:29. doi: 10.1186/s12898-018-0185-9 (PMC6117883; doi:10.1186/s12898-018-0185-9)
Supplement: Supplementary file 5 — Additional file 5: Table S3. Mean environmental variables during CO2 flux measurements. Table S4. Mean summer and winter surface temperature and snow depth. [file 12898_2018_185_MOESM5_ESM.pdf]

Additional file 5
For
Experimental herbivore exclusion, shrub introduction, and carbon sequestration in alpine plant communities

Author names: Mia Vedel Sørensen*, Bente Jessen Graae, Dagmar Hagen, Brian J. Enquist, Kristin Odden Nystuen, Richard Strimbeck

***Corresponding author**: Mia Vedel Sørensen, email: miavedelsorensen@gmail.com

Table S3: Environmental variables during CO_2 flux measurements: Moisture = soil moisture (%), T_{air} = air temperature inside chamber (°C), $T_{surface}$ = temperature at 1 cm depth, T_{soil} = soil temperature at 8 cm depth (°C), PAR = Photosynthetic Active Radiation inside the chamber (µmol m^{-2} s^{-1}). For treatments with and without Exclosure and Transplant in alpine *Empetrum*-heath, meadow and *Salix*-shrub communities in Dovre Mountains, central Norway. Estimates and ± 95 % confidence intervals (CI) are from linear mixed effects models fitted with community, Exclosure, Transplant and the interaction of the three as fixed effects, and plot as random effect.
In the shrub community moisture was significantly different with and without exclosure ($F_{1, 30}$ =4.35, *p* = 0.046, ANOVA) as it was drier within the exclosures (*p = 0.037*, Tukey). Air temperature was also significantly different with and without exclosure ($F_{1, 58}$ =5.50, *p* = 0.022, ANOVA) as it was cooler inside the exclosures (*p = 0.019*, Tukey). In the meadow the surface temperature was significantly different with and without exclosure ($F_{1, 30}$ = 6.2, *p* = 0.018, ANOVA) as it was cooler inside the exclosures (*p = 0.013*, Tukey).

		**Exclosure** No	**Transplant** No	**Exclosure** No	**Transplant** Yes	**Exclosure** Yes	**Transplant** No	**Exclosure** Yes	**Transplant** Yes
		**Estimate**	**CI**	**Estimate**	**CI**	**Estimate**	**CI**	**Estimate**	**CI**
Moisture	Heath	19.4	± 6.5	18.5	± 6.3	20.1	± 6.3	19.0	± 6.3
	Meadow	27.4	± 5.2	26.3	± 4.8	27.2	± 4.8	27.2	± 4.8
	Shrub	37.9	± 5.4	28.6	± 5.1	25.9	± 5.0	22.5	± 4.9
Tair	Heath	21.5	± 1.9	21.3	± 2.0	20.9	± 2.0	21.6	± 2.0
	Meadow	21.9	± 1.9	21.6	± 1.7	22.0	± 1.7	21.6	± 1.7
	Shrub	21.1	± 1.9	21.4	± 1.8	19.4	± 1.8	19.3	± 1.7
Tsurface	Heath	15.1	± 1.5	15.5	± 1.7	13.6	± 1.5	13.6	± 1.5
	Meadow	14.1	± 1.5	13.9	± 1.4	12.7	± 1.4	12.3	± 1.4
	Shrub	11.1	± 1.5	10.9	± 1.4	10.0	± 1.4	10.6	± 1.4
Tsoil	Heath	10.7	± 0.8	9.8	± 0.8	10.1	± 0.8	10.3	± 0.8
	Meadow	10.1	± 0.7	9.7	± 0.6	9.2	± 0.6	9.5	± 0.6
	Shrub	8.4	± 0.8	8.9	± 0.7	8.5	± 0.7	8.5	± 0.7
PAR	Heath	609	± 113	662	± 123	587	± 118	671	± 118
	Meadow	539	± 109	599	± 96	610	± 96	621	± 96
	Shrub	607	± 113	580	± 105	576	± 102	544	± 99

Table S4: Temperature surface ($_{Tsurface}$) summer and winter means ± SD across 2014 and 2015 and snow depth means ± SD in 2015, for treatments with and without Exclosure and Transplant in alpine *Empetrum*-heath, meadow and *Salix*-shrub communities in Dovre Mountains, central Norway. Summer reflects the warmest months (July and August) and winter the coldest months (January and February). The surface temperature was measured daily every four hours. Statistical differences were tested within each community, for $T_{surface}$ with ANOVA on linear mixed models where exclosure, transplant, and year were fixed factors, and plot was random factor. Snow depth were measured 4 times per plot in early March 2015. Statistical differences were tested within each community with ANOVA on a linear model. In meadow and heath communities $T_{surface}$ summer was statistically lower with exclosures ($p < 0.05$, Tukey), but there were no difference in the shrub community. In the meadow community $T_{surface}$ winter was significantly higher with exclosures, but there were no significant differences in the heath and shrub communities. Snow depth was significantly ($p < 0.05$, Tukey) deeper within the exclosures in the heath, but there were no significant differences with treatments in the meadow and shrub communities.

Community	Exclosure	Transplant	$T_{surface}$ summer (°C)	$T_{surface}$ winter (°C)	Snow depth (cm)
Heath	no	no	11.93 ± 0.54	-3.19 ± 0.54	0 ± 0
	no	yes	11.90 ± 0.57	-2.89 ± 0.36	0 ± 0
	Yes	no	11.39 ± 0.20	-2.83 ± 0.47	0.96 ± 1.77
	Yes	yes	11.51 ± 0.32	-2.98 ± 0.27	1.08 ± 1.72
Meadow	no	no	11.50 ± 0.64	-1.18 ± 0.27	38.34 ± 4.41
	no	yes	11.54 ± 0.43	-1.23 ± 0.34	42.31 ± 7.22
	yes	no	11.08 ± 0.43	-0.88 ± 0.37	41.41 ± 8.42
	yes	yes	10.98 ± 0.49	-0.86 ± 0.30	47.53 ± 10.52
Shrub	no	no	10.11 ± 0.80	-0.92 ± 0.35	50.88 ± 23.61
	no	yes	10.11 ± 0.57	-0.72 ± 0.39	49.84 ± 21.16
	yes	no	9.79 ± 0.71	-0.58 ± 0.14	49.44 ± 13.20
	yes	yes	9.72 ± 0.50	-0.71 ± 0.32	47.97 ± 12.43
